# Response to Letter to the Editor from Berends et al: “Approach to the Patient: Perioperative Management of the Patient With Pheochromocytoma or Sympathetic Paraganglioma”

**DOI:** 10.1210/clinem/dgaa602

**Published:** 2020-09-07

**Authors:** Annika M A Berends, Michiel N Kerstens, Jacques W M Lenders, Henri J L M Timmers

**Affiliations:** 1 Department of Endocrinology, University of Groningen, University Medical Center Groningen, Groningen, the Netherlands; 2 Department of Internal Medicine, Radboud University Medical Center, Nijmegen, the Netherlands; 3 Department of Medicine III, University Hospital Carl Gustav Carus, Technical University Dresden, Dresden, Germany

**To the Editor,**

We thank Dr Yu for his comments in response to our article. According to Dr Yu, we did not sufficiently address 2 misconceptions on perioperative management in patients with a pheochromocytoma. He states that we suggested that control of hypertension is more important than prevention of cardiovascular complications, based on the order in which we mentioned these goals. These goals, however, were described in random order and we did not mean to imply that one was more important than the other. Fluctuations in blood pressure and heart rate are nevertheless common in pheochromocytoma, may have deleterious effects per se, and are amenable to medical management. We do agree with Dr Yu that normotension does not preclude the occurrence of cardiovascular complications, an important message to which we have also brought attention.

In response to the alleged second misconception in our paper, Dr Yu suggests that the perioperative management should be more individualized. We agree that in general, any treatment should be tailored to the patient. In fact, when aiming at predefined perioperative hemodynamic targets as we suggest, this automatically translates into an individualized approach to the selection of drugs and doses. Unfortunately, however, it is still largely unknown which specific preoperative factors reliably predict a person’s perioperative risk and the ensuing optimal treatment. His statement that preoperative echocardiography and 24-hour ambulatory blood pressure monitoring is unnecessary in patients harboring a small pheochromocytoma (ie, < 3 cm) who are physically fit implies that pheochromocytoma/paraganglioma (PPGL)-related cardiovascular complications are negligible under these circumstances. The notion of skipping these routine and noninvasive investigations is, however, not corroborated by the literature that was cited. For example, the study by Yu et al demonstrated that 17% of patients with a tumor smaller than 3 cm also developed severe cardiovascular complications during unrelated procedures (1). In addition, in the series by Chen and colleagues, a smaller tumor size was not accompanied with a lower rate of complications (2). Moreover, the clinical picture of a PPGL-induced cardiomyopathy is often atypical. Only a minority presents with classical cardiac symptoms, and absence of cardiovascular risk factors or a young age does not protect against the development of this form of cardiomyopathy (3-5). Furthermore, the assumption that tumor size strongly influences the risk for cardiac complications is not supported by a high level of evidence, as was also shown in the review by Shen and Yu (6). Of relevance, preoperative demonstration of abnormalities on electrocardiogram or echocardiography does predict an increased risk for cardiac complications (6).

In conclusion, a small PPGL size or the absence of symptoms does not reliably predict a low perioperative risk. We do agree that the controversy with respect to the clinical value of presurgical treatment with α-adrenoreceptor blockers in modern-day medicine could be resolved only by a well-designed placebo-controlled trial. Until then, we recommend following the Endocrine Society’s guideline that all patients with a PPGL, regardless of blood pressure and tumor size, should undergo preoperative evaluation of cardiac function and blood pressure and receive presurgical treatment with α-adrenoreceptor blockers (7).

## Additional Information

***Disclosure Summary:*** The authors have nothing to disclose.
